# Resident-Performed Coronary Artery Bypass Grafting Using Bilateral Internal Mammary Arteries: Clarifying Methodological Gaps

**DOI:** 10.1093/icvts/ivaf199

**Published:** 2025-08-20

**Authors:** Pradeep Narayan

**Affiliations:** Department of Cardiac Surgery, Rabindranath Tagore International Institute of Cardiac Sciences, Narayana Health, Mukundapur, Kolkata 700099, India

The authors have to be congratulated for undertaking an extremely relevant topic for their research in the area of coronary artery bypass grafting using bilateral internal artery grafting.^1^ They show that BIMA grafting by residents can be accomplished safely under supervision. Previous studies have shown similar findings.^2^ However, few important points need to be highlighted.

The study does not detail the prior surgical experience in terms of Coronary Artery Bypass Grafting (CABG) procedures or anastomoses each trainee had completed beforehand, nor how many residents were involved. Thus, it is unclear whether the reported outcomes reflect broad trainee competency or the performance of a select few. Over the 12-year study period, 431 BIMA procedures were performed by residents, averaging 36 cases/year or 3/month. The manuscript provides no information on how these cases were distributed across trainees. It is therefore impossible to determine whether or not most procedures were concentrated among a small number of more proficient trainees, and the outcomes may simply reflect the skill of a few capable trainees and thus cannot be generalized to the wider trainee population.

The degree of staff involvement during resident-performed surgeries is also not quantified. It is unclear if, in technically demanding situations, the anastomosis was completed by staff surgeons. It is also important to note that 52.5% of patients in the staff surgeon group received ≥3 grafts, compared to only 43.2% in the resident group. This strongly suggests a selection bias, with patients requiring fewer grafts being preferentially allocated to residents. Given the resident group also had lower rates of composite grafting and fewer anastomoses to the Right Coronary Artery (RCA) territory, it is likely that both case complexity and disease burden were lower in this group.

While there was no difference in outcome at 30 days, it has to be asked, especially given the low incidence of major adverse events such as mortality and stroke, if the study was powered to detect these differences in the first place. Finally, the 2 groups differed significantly in baseline characteristics, including EuroSCORE II, left ventricular function, coronary disease severity, and urgency of surgery, and propensity matching may have been useful.

While the authors’ efforts are commendable, the manuscript’s impact and interpretability would have been significantly enhanced by the inclusion of the aforementioned details.
